# Effects of Immunonutrition on Cancer Patients Undergoing Surgery: A Scoping Review

**DOI:** 10.3390/nu15071776

**Published:** 2023-04-05

**Authors:** Katherine García-Malpartida, Carmen Aragón-Valera, Francisco Botella-Romero, María Julia Ocón-Bretón, Juan J. López-Gómez

**Affiliations:** 1 Endocrinology and Nutrition Department, Hospital Universitario y Politécnico La Fe, 46026 Valencia, Spain; 2 School of Health Sciences, Universidad Cardenal Herrera-CEU, CEU Universities, Calle Grecia 31, 12006 Castellón, Spain; 3 Hospital Universitario Fundación Jiménez Díaz, 28040 Madrid, Spain; carmen_arval@telefonica.net; 4 Coordinador Comité Gestor Área de Nutrición, Sociedad Española Endocrinología y Nutrición (SEEN); 28001 Madrid, Spain; botellaromero@gmail.com; 5 Nutrition Department, Hospital Clínico Universitario Lozano Blesa, 50009 Zaragoza, Spain; mjocon@salud.aragon.es; 6 Endocrinology and Nutrition Department, Hospital Clínico Universitario de Valladolid, 47003 Valladolid, Spain; 7 Centro de Investigación Endocrinología y Nutrición, Universidad de Valladolid, 47003 Valladolid, Spain

**Keywords:** oncological surgery, immunonutrition, cancer, complications, mortality

## Abstract

Introduction: There is a large body of evidence about immunonutrition formulas; however, there are still doubts about their usefulness in routine clinical practice as compared with standard formulas. In the age of personalized medicine, new studies appear every year regarding several types of patients; therefore, an updated point of view on these formulas is necessary. Methods: The Embase database was searched from 2016 to 14 March 2022. Our criteria were articles published in English and Spanish. The evidence quality was evaluated using GRADEpro, and the review was developed according to the PRISMA statement. Results: In this review, a total of 65 unique records were retrieved; however, 36 articles did not meet the inclusion criteria and were thus excluded. In total, 29 articles were included in the final analysis. In the last few years, many meta-analyses have attempted to identify additional existing studies of surgical patients with certain pathologies, mainly oncological patients. Immunonutrition prior to oncological surgery was shown to cause a decrease in inflammatory markers in most of the studies, and the main clinical events that changed were the infectious complications after surgery. The length of stay and mortality data are controversial due to the specific risk factors associated with these events. Conclusions: The use of immunonutrition in patients who have undergone oncological surgery decreases the levels of inflammatory markers and infectious postoperative complications in almost all localizations. However, more studies are needed to assess the use of immunonutrition based on Enhanced Recovery After Surgery (ERAS) protocols.

## 1. Introduction

Disease-related malnutrition (DRM) is a condition observed in some types of patients that is defined as “a specific type of malnutrition caused by a concomitant disease” [1]. Patients who undergo a major surgery have an increased risk of DRM and complications derived from it [2]. This pathology is a consequence of a decrease in energy–protein intake with an increase in chronic or acute inflammation [1]. Medical Nutrition Therapy (MNT) must be used to optimize energy–protein intake; however, improving inflammatory profiles remains a challenge. Immunonutrition was developed in an attempt to control this mechanism and its possible associated comorbidities in the genesis of malnutrition.

Oncological pathology has an increasingly high prevalence and influences the quality of life of patients affected by it. A patient with oncological pathology of any type has an increased risk of malnutrition. Between 15 and 40% of cancer patients present some degree of malnutrition at disease diagnosis. This condition worsens with disease progression, with 80% of patients affected by malnutrition in the advanced stages of cancer. The main characteristics of cancer patients are the existence of chronic inflammation due to the disease and its treatment [3]. Oncological patients may need medical nutritional treatment to achieve an adequate clinical status prior to surgery [4].

Immunonutrition is a type of artificial nutrition based on the use of some types of macro- or micronutrients. These immunonutrients can modulate the inflammatory response and influence disease evolution. Some pathologies, such as sepsis, surgery, trauma, and burns, are characterized by an uncontrolled inflammation and/or immune suppression associated with tissue damage and an increase in infection and cardiovascular dysfunction [5]. Some immunonutrients, such as arginine, nucleotides, and glutamine, can reduce the inflammatory response, and other immunonutrients, such as omega-3 fatty acids, can enhance the immune response [6].

For this reason, the use of artificial nutrition enhanced with some immunonutrients is postulated as a method of nutritional treatment. This treatment could be an option used to achieve nutritional requirements and modulate the immune response in patients with specific pathologies or cancer or prior to surgery. However, the evidence-based recommendations of these types of formulas in clinical practice are scarce and limited to specific populations.

In critical patients with mechanical ventilation, some large studies, such as RE-DOXs and MetaPLUS, showed that the use of enteral formulas with immunonutrients, such as antioxidants or glutamine, did not improve complications and other clinical outcomes, and the use of this type of nutrition even increased mortality rates [6,7]. These controversial results make it difficult to determine which patients can benefit from the use of these nutrients and when they should be delivered. The best results have been observed in trauma and medical patients undergoing elective surgery, though not in situations of sepsis [8].

In surgical patients, the use of glutamine or arginine in an isolated form has not shown a clinical effect on the occurrence of events after surgery [2]. The use of omega-3 can reduce inflammatory markers in patients undergoing elective gastrointestinal surgery [9]; however, the evidence on clinical events is controversial. Despite this, the use of immunonutrient-enriched enteral formulas can help to address clinical events in some types of abdominal surgeries, specifically in upper-gastrointestinal-tract cancer, and the ESPEN (European Society of Clinical Nutrition and Metabolism) guidelines on surgery recommend these formulas for these patients [2]. The effect is striking in those patients at risk of malnutrition or with disease-related malnutrition; therefore, there are some concerns about the effects of nutritional treatment based on immunonutrient enrichment.

There is high variability in the development of immunonutrition formulas, with those enriched in amino acids, such as glutamine and arginine, and those with high omega-3 fatty acid contents provided to surgical patients. On the other hand, there are formulas with higher doses of omega-3 fatty acids and reduced amino acid contents aimed towards cancer patients. Some immunonutrition formulas have a reduced carbohydrate content and a high fat content (especially omega-3 fatty acids), and they are used in cases of Adult Respiratory Distress Syndrome (ARDS). The current guidelines mention immunonutrition as a type of treatment for some pathologies; however, there is no clear definition of immunonutrition content or instauration timing [2].

There is a large body of evidence about this type of enteral formula; however, there are still doubts about its usefulness in routine clinical practice as compared with standard formulas. This situation may be due to the fact that patients with cancer undergoing surgery have heterogeneous characteristics, such as the type of cancer, its aggressivity, type of surgery, and the need for chemotherapy or radiotherapy prior to intervention. These features can influence surgery events and can mask the effects of immunonutrition. In the age of personalized medicine, new studies of several types of patients are appearing every year; therefore, an updated point of view on these formulas is necessary. The purpose of this review was to evaluate the use, indications, and effects of these formulas in oncologic surgical patients in real time and to identify the types of patients who can benefit from enteral immunonutrition.

## 2. Materials and Methods

The Embase database was searched from 2016 to 14 March 2022, and the following keywords were used: “immunonutrition”, “surgery”, “preoperative period”, “postoperative care”, “enhanced recovery after surgery”, “fast track surgery”, “postoperative complication”, “postoperative morbidity”, “surgical mortality”, “hospital readmission”, “malignant neoplasm”, “cancer surgery”, “traumatic brain injury”, “sepsis” “acutely ill patient”, “burn patient”, “stem cell transplantation”, “bone marrow transplantation”, “inflammatory bowel disease”, “chronic inflammation”, “sarcopenia”, “skeletal muscle mass”, “functional status”, “cachexia”, “malnutrition”, “body composition”, “bioelec-trical impedance”, “phase angle”, “echography”, “computer assisted tomography”, “L3 muscle”, “dual energy x ray absorptiometry”, “randomized controlled trial”, “systematic review”, “meta-analysis”, and “human”. Our criteria were articles published in English and Spanish (Table 1).

In this analysis, the clinical outcomes included postoperative infectious complications, postoperative deep venous thrombosis, the incidence of pulmonary infection, changes in body weight, mortality, the length of hospitalization, systemic inflammatory response syndrome, incision infection, and relevant T cell subsets, which included CD3+, CD4+, and CD8+.

The selected articles included studies of preoperative or postoperative immunonutrition in patients with cancer or who received oncological surgery. The type of article had to be a systematic review, meta-analysis, or randomized clinical trial (RCT).

The following articles were excluded: those documenting patients undergoing non-oncological surgery; non-randomized clinical trials; and general articles with no relevant information or experimental data in cases where we were unable to acquire primary data and essential information from the authors. The titles and abstracts were used to exclude clearly irrelevant articles.

The inclusion and exclusion criteria were applied to the articles, and these articles were distributed between authors according to the type of surgery. Each article was peer-reviewed by all the authors and added to the GRADEpro tool. A second GRADE review was performed by an independent author to assess the value of the research and the evidence summary.

**Figure 1:**
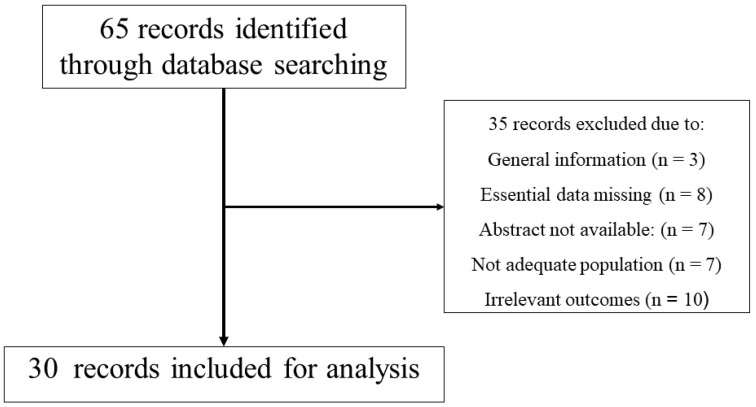
Flow Chart.

In this review, a total of 65 unique records were retrieved, while 30 articles did not meet the inclusion criteria and were thus excluded. In total, 35 articles were included in the final analysis (Figure 1).

The review was assessed using the PRISMA statement. The base was registered in the OSF repository (https://osf.io/dashboard, accessed on 13 March 2023) with a register code and link https://osf.io/mxz95 (accessed on 13 March 2023) (Supplementary Table S1).

The quality of evidence was evaluated using the GRADEpro tool on the webpage https://www.gradepro.org/ (accessed on 13 March 2023). The articles were ranked from high to very low considering the risk of bias, study design, sample size, indirect evidence, or lack of precision. The quality of the studies, assessed by GRADEpro, is shown in the Supplementary Data (Supplementary Tables S2–S8).

## 3. Results

We selected 30 articles on the research topic of immunonutrition as an intervention in some surgical procedures. The papers were revised considering the types of surgical patients: general oncologic patients, head and neck cancer surgery, hepatic surgery, bladder surgery, colorectal surgery, pancreatic surgery, and esophagogastric surgery.

The results from 14 reviewed randomized clinical trials are shown in Supplementary Table S9.

### 3.1. Oncologic Patient

A meta-analysis conducted in 2020, including 5983 patients, compared immunonutrition (oral, enteral, and parenteral, including at least one of the following nutrients: arginine, glutamine, omega-3 fatty acids, and/or nucleotides) with conventional nutrition and fluid therapy in the perioperative period among cancer patients who underwent surgery. The intervention group showed a reduction in the total number of infectious complications (risk ratio (RR) 0.71 (0.64–0.79)), wound infections (sample size (*n*) = 4788, RR 0.72 (0.60–0.87)), respiratory infections (*n* = 4919, RR 0.70 (0.59–0.84), urinary tract infection (*n* = 3686, RR 0.69 (0.51–0.91), anastomotic dehiscence (*n* = 3329, RR 0.70 (0.53–0.91)), and hospital stay (−2.12 (−2.72–−1.52) days). There were no differences in the number of episodes of sepsis (*n* = 2322) or overall mortality [10].

Another meta-analysis published in the same year analyzed the benefits of presurgical oral immunonutrition in cancer patients undergoing surgery. It included 2159 patients from 22 studies. Immunonutrition was administered from 30 to 5 days before surgery and was allowed to continue after surgery, administered by the oral or enteral route. Immunonutrition reduced the total numbers of infectious complications (*n* = 2068, RR 0.58 (0.48–0.70) and surgical site infection (*n* = 1958, RR 0.65 (0.50–0.85)). There were no differences in mortality (*n* = 1641) [11].

A narrative review of 40 studies (5 in humans and the rest in animals) on the effects of key amino acids, such as leucine, beta-hydroxy-beta-methylbutyrate (HMB), arginine, glutamine, and creatine, on cancer-induced sarcopenia and cachexia concluded that although the administration of amino acids produced and increased protein synthesis in rodents, a gain in muscle mass was not demonstrated in cancer patients [12]. The main results are shown in Table 2.

### 3.2. Head and Neck Cancer Surgery

A Cochrane review developed by Howes et al. studied the effects of immunonutrition compared with standard feeding on patients undergoing surgery for head and neck cancer. It included 19 RCTs with 1099 participants aged between 47 and 66 years. There was no evidence of differences in the length of stay (mean difference −2.5 days (−5.11–0.12)) or the effect of immunonutrition on wound infection (RR 0.94 (0.70–1.26)). Nevertheless, fistula formation was reduced using the immunonutrition formula (RR 0.48 (0.27–0.85)). There were no differences in feed tolerance between treatments (RR 1.33 (0.86–2.06)). Mortality did not differ between groups (RR 1.33 (0.48–3.66)) [13].

A phase II RCT conducted by Dechaphunkul et al. studied patients undergoing concurrent chemoradiation and randomly assigned the patients to immunonutrition (omega-3 fatty acids, arginine, dietary nucleotides, and soluble fiber) (55 patients) or a standard formula (isocaloric and isoprotein formula) (55 patients) 5 days before each chemotherapy session. There was no difference in the number of patients with grade 3–4 mucositis between the groups (62% vs. 67%; *p* = 0.690) [14]. The main results are shown in Table 3.

### 3.3. Hepatic Surgery

In a 2020 meta-analysis that included 966 patients undergoing hepatectomy from 9 studies, the use of immunonutrition or omega-3 fatty acids (pre- or post-surgery) was associated with a decrease in the numbers of postoperative complications (RR 0.57 (0.34–0.95)), total infections (RR 0.53 (0.37–0.75)), wound infection (RR 0.50 (0.28–0.89)), pneumonia (RR 0.60 (0.32–1.12)), urinary tract infections (RR 1.30 (0.55–3.08)), liver failure (RR 0.54 (0.23–1.24)), mortality (RR 0.69 (0.26–1.83), and length of stay (−3.80 (−6.59–−1.02) days) [15]. A previous meta-analysis from 2017 on the same type of patients obtained similar results regarding postoperative complications and infections, with no differences in mortality [16].

A multicenter study conducted in France that included 399 patients compared the use of immunonutrition with an isocaloric formula based on the same protein intake 7 days before liver resection surgery for non-cirrhotic cancer. This intervention did not reduce the 30-day morbidity or mean hospital stay [17]. Uno et al in 2016 show similar results [18].(Table 4).

### 3.4. Bladder Surgery

We only found two publications regarding patients who underwent radical cystectomy for bladder cancer. Hamilton-Reeves et al. randomized 29 patients who received 3 bricks per day of an oral nutritional supplement (ONS) with immunonutrition (14 patients) or a standard ONS (15 patients) 5 days before and 5 days after radical cystectomy. The authors observed favorable immunological changes [19] and a 33% reduction in postoperative complications in the group receiving immunonutrition at 90 days (RR 0.31, (0.08–1.23); *p* = 0.060), without observing significant differences in the length of hospital stay between the two groups [20] (Table 5).

In a Systematic Review by Alan et al and a Cochrane review, the authors assigned a low quality of evidence to the study due to imprecision errors and the small sample size, which limited the study findings [21,22].

### 3.5. Colorectal Surgery

In total, one meta-analysis and four randomized studies (two of which analyzed patients included in Enhanced Recovery After Surgery (ERAS) programs) were collected for patients undergoing surgery for colorectal cancer (Table 6).

Lee et al. randomized 176 patients who received either 400 mL of an immunonutrition ONS daily (88 patients) or a regular diet (88 patients). The authors found no differences in relation to the numbers of infectious complications (17.7% vs. 15.0%; *p* = 0.751), total postoperative complications (31.6% vs. 29.3%; *p* = 0.743), prolongation of hospital stay (7.6 (2.5) vs. 7.4 (2.3) days; *p*= 0.635), or changes in body weight between groups [23].

In another pilot study carried out among 26 patients, who were randomized to receive treatment 14 days before surgery (an ONS with immunonutrition versus a standard ONS), it was shown that immunonutrition can influence the expression of inflammatory cytokines (TNF-alfa, IL8, or chemokines) and the infiltration of leukocytes into the tumor tissue [24].

We analyzed two Spanish multicenter studies published by Moya et al., in which patients undergoing surgery for colorectal cancer, who were included in an ERAS program, were evaluated. The protocols of the two studies were very similar, as were their results. In one study, 264 normally nourished patients were randomized to receive 200 mL of an immunonutrition ONS daily in addition to their regular diet for 7 days before and 5 days after surgery. The control group received 400 mL per day of a hypercaloric and hyperproteic ONS together with their diet for the same period of time [25]. The second study was carried out with 128 normally nourished patients who underwent laparoscopy and were randomized to consume 400 mL per day of an immunonutrition ONS in addition to their regular diet for 7 days before and 5 days after surgery. The difference in this study was that the patients in the control group received dietary advice alone [26]. In the study with the largest sample size, the authors observed a significant decrease in the number of infectious complications (23.8 vs. 10,7%; *p* < 0.0007), and in both studies, IMN significantly reduced the cases of wound infection (16.4% vs. 5,7%; *p* = 0.0008; and 11.50 vs. 0.00%; *p* = 0.006), without showing significant differences in relation to the length of stay [26,27].

Xu et al. carried out a systematic review and meta-analysis of six studies (four prior to 2014 and the remaining two corresponding to the two articles published by Moya et al.) to analyze the effect of immunonutrition on patients with colorectal cancer undergoing elective surgery. The conclusions noted by the authors were that immunonutrition can reduce the length of hospital stay (OR 2.53 (1.29–2.41)) and number of infectious complications (OR 0.33; (0.21–0.53)) [27].

### 3.6. Esophagogastric Surgery

In the literature review, we found 10 articles on esophagogastric surgery, 4 clinical trials, and 6 systematic reviews/meta-analyses (Table 7). Another clinical trial conducted by Yu et al. evaluated the effects of two immunomodulatory formulas (arginine, nucleotides and omega-3, or RNA and omega-3) on nutritional status and functional capacity in 202 patients who were candidates for major oncological surgery for gastrointestinal cancer. The patients who received the arginine-containing formula experienced a lower decrease in prealbumin on the eighth postoperative day (*n* = 102 18 vs. 16.7 mg/dL; *p* = 0.045), and they also lost less weight (1.63 vs. 244 kg; *p* = 0.009) and presented more frequently an Eastern Cooperative Oncology Group (ECOG) grade <2 (93 vs. 82%; *p* = 0.022) [28].

The meta-analysis conducted by Cao et al. aimed to verify whether preoperative immunonutrition improves the postoperative results of complications of patients undergoing esophageal surgery and their in-hospital mortality in the postoperative period. For this purpose, 15 randomized clinical trials (RCTs) (1864 patients) were analyzed to compare a standard nutritional intervention versus immunonutrition. This review found significant differences showing fewer infectious complications (odds ratio (OR) 0.51 (0.26–0.98)) and a shorter length of stay (mean difference = −2.10 day (−3.72–−0.47)), with no significant in-hospital mortality (OR = 1.03 (0.41–2.61)), total complications (OR = 0.76 (0.52–1.11)), or anastomotic leaks (OR = 1.05 (0.69–1.58)). There is no information on the presurgical treatment [29].

Cheng et al. collected seven studies (583 patients) on patients who underwent gastrectomy for gastric cancer, and they evaluated the clinical, biochemical, and immune results after the use of immunonutrition in the postoperative period. They found improvements in biochemical and immunity parameters (increased CD4+ (SMD = 0.99; 95% CI, 0.65–1.33; *p* < 0.00001), CD4+/CD8+ (SMD = 0.34; 95% CI, 0.02–0.67; *p* = 0.04), IgM (SMD = 1.15; 95% CI, 0.11–2.20; *p* = 0.03), IgG (SMD = 0.98; 95% CI, 0.55–1.42; *p* < 0.0001), lymphocytes (SMD = 0.69; 95% CI, 0.32–1.06; *p* = 0.0003) and prealbumin (SMD = 0.73; 95% CI, 0.33–1.14; *p* = 0.0004). The clinical variables, such as systemic inflammatory response syndrome (−0.89 days (−1.4–−0.39); *p* = 0.005) and postoperative complications (RR 0.29 (0.14–0.60); *p* = 0.001), were significantly reduced in the group of patients for whom immunonutrition was postoperatively maintained for >7 days [30].

Niu et al. presented a systematic review of 25 RCTs, of which 16 were included in their meta-analysis, finding a significant reduction in the number of surgical infections and length of hospital stay but not in other clinical parameters, although this did not include the number of patients subject to each statistical analysis [31].

The study conducted by Adiamah et al. analyzed the long-term survival (>20 years) of 108 patients (54 in each group) who were included in a previous RCT in 3 hospitals in the United Kingdom (UK) to determine whether supplementation with arginine, as part of the immunonutrition formula administered for 15 days through jejunostomy during the postoperative period, in esophagogastric or pancreatic cancer patients improves survival compared with isocaloric and isoprotein nutrition, used in the control group. No significant differences in long-term survival were found [32].

This study agrees with the meta-analysis conducted by Li XK et al. based on 7 studies (6 of them in Japan) with a total of 320 patients who underwent esophagectomy, in which highly heterogeneous regimens were used (pre- and postoperative ONS; complete enteral nutrition) for immunonutrition vs. a standard formula. No changes were found in any of the clinical variables [33].

Zhuo et al. evaluated the effects of immunomodulatory versus standard formulas on patients who underwent esophagectomy. No differences were found in the number of infectious complications or complications associated with surgery, as follows: anastomotic fistula: 9% vs. 10.9% (OR = 0.75 (0.43–1.31); *p* = 0.32); surgical wound infections: 8.8 vs. 9.8% (OR = 0.99 (0.49–2.00); *p* = 0.98); and lung infections: 18.7% vs. 14.6% (OR = 1.04 (0.65–1.65); *p* = 0.88). The appearance of sepsis was evaluated in three studies, and there were no differences (6.1% vs. 5.5% OR = 0.92 (0.42–2.02); *p* = 0.84) [34]. On this basis, Li XT developed an RCT that included 112 randomized patients receiving ONS (750 kcal/day + oral diet) of an immunonutrition formula versus isocaloric and isoproteic standard formulas for 7 days prior to esophagectomy and 30 days after surgery. This study only found changes in immunological parameters (lower percentages of CD8/CD3, *p* = 0.005; a higher CD8/CD3 ratio, *p* = 0.004). Likewise, the serum IgM levels were higher in the group postoperatively treated with immunonutrition on both day 3 and day 7; however, there were no significant differences in the clinical variables, disease progression, or overall survival [35].

In a similar study conducted by Kanekiyo et al., 40 patients were randomly assigned to receive supplementation with immunonutrition vs. standard nutrition together with their normal diet for 7 days prior to surgery and via jejunostomy, as the complete formula, for 7 days in the postoperative period, and they showed improvements in the retinol-binding protein (RBP) levels, with an increase from the day before surgery to 14 days in the postoperative period. Furthermore, the numbers of infectious complications were significantly lower. However, there were no significant differences in the number of days spent in the ICU or on the ward, progression, or 5-year survival [36].

Song et al. performed a study on patients undergoing oncological gastrectomy (*n* = 840, 426 in the treatment group). Its main objective was to evaluate the efficacy of different types of immunomodulatory formulas against placebo in the prevention of postoperative complications and to determine the influence of the mean stay. The types of formulas analyzed were arginine + RNA, arginine + RNA + omega-3 fatty acids, and arginine + glutamine and arginine + glutamine + omega-3 fatty acids. The four immunomodulatory formulas were superior to the standard in preventing infectious complications; however, there were no differences in the rest of the complications. In the comparison of specific versus standard formulas, arginine + RNA + omega-3 and arginine + glutamine + omega-3 were superior (RR 0.27, 95% CI 0.12–0.49 and RR 0.22, 95% CI 0.02–0.84). Regarding non-infectious complications, there were no differences from the standard formula in general or for each of the immunonutrition subtypes. In terms of the length of stay, the immunomodulatory formulas were associated with a shorter stay, and discriminating by the type of formula, a reduction was observed for arginine + RNA + omega-3 (MD −0.58, 95% CI −0.98—0.17) and arginine + glutamine + omega-3 (MD −0.69, 95% CI −1.22—0.17). In the comparison between formulas, the potential usefulness of arginine + RNA + omega-3 was observed when compared with the rest of the formulas [37].

Metaanalysis from Wong et al. showed a decrease in wound infections and LOS in patients with upper gastrointestinal surgery [38].

### 3.7. Pancreatic Surgery

In recent years, there have been three meta-analyses of pancreas cancer and its surgery: two of them analyzed pancreatoduodenectomy, and one investigated pancreatic cancer surgery (Table 8).

Takagi et al. evaluated partial pancreatoduodenectomy and observed a reduction in the total number of complications and infections; however, there were no effects on major complications, mortality, fistula, or gastric emptying [39]. Nevertheless, Guan et al. analyzed pancreatoduodenectomy and observed reductions in only the number of infectious complications (RR 0.58 (0.37–0.92) and length of hospital stay (MD −1.79, (−3.40–0.18), with no differences in overall complications [40]. On the other hand, Yang et al. studied patients with cancer without differentiating the surgical technique and also observed reductions in the number of infectious complications (RR = 0.47 (0.23–0.94) and length of hospital stay (MD = −1.9 (−3.78–0.02) [41].

## 4. Discussion

This review was created to identify indications of surgical patients who could benefit from immunonutrition. In the last few years, many meta-analyses have attempted to identify additional existing studies of surgical patients with certain pathologies, mainly oncological patients. Immunonutrition treatments prior to oncological surgery showed a decrease in inflammatory markers in most of the studies, and the main clinical events that changed were the infectious complications after surgery. The length of stay and mortality data are controversial due to the specific risk factors associated with these events (Table 9).

Immunonutrition treatments have been shown to have considerable effects on patients with oncologic pathologies undergoing surgery. Most of the clinical guidelines propose immunonutrition or immunonutrients, such as omega-3 fatty acids, as a method of enteral nutrition for oncologic patients at risk of malnutrition [4] or surgical patients, mainly those with oncologic abdominal pathology [2]. Our review confirms this. Our meta-analysis analyzed a selection of general oncological surgical studies which compared immunonutrition with conventional nutrition, showing reductions in clinical features, especially infectious complications and the hospital stay [10,11]. However, there is no evidence to suggest that these formulas can reduce mortality or sepsis among oncological patients. In these patients, mortality is influenced by many conditions, such as muscle mass [42], age, previous comorbidities [43], and previous history of toxic consumption, such as alcohol or smoking [44]. These conditions cannot always be changed through the intake of immunonutrition.

Another issue that must be considered regarding patients with cancer is the decrease in muscle mass and the presence of caquexia. The effects of these formulas in these contexts are still unclear, and the positive effects of immunonutrients, including amino acids such as leucine, arginine, glutamine, and their metabolites, are only evidenced in rodents; there is not enough evidence in humans. The effects of using amino acids, such as leucine, or branched amino acids have been shown to include changes in muscle mass and function in patients with sarcopenia; however, the effect of using formulas of this type of amino acids in cancer patients is still unclear [45].

Immunonutrition treatments for head and neck cancer patients are controversial. Most of the studies on the currently developed treatments have shown a decrease in the development of tracheoesophagic fistula after surgery; however, there is no improvement in the length of stay or mortality. In the last few years, there has been a lack of evidence regarding the effects of previous chemotherapy or radiation treatments. Only one study evaluated the effect of immunonutrition on patients undergoing chemoradiation, and it did not show any differences in morbidity (grade 3–4 mucositis) [14]. At this point, studies on head and neck cancer are scarce, and those that do exist have multiple bias, as the Cochrane review mentioned [13].

In patients undergoing hepatectomy, two recent meta-analyses showed benefits in postoperative comorbidity, mortality, and the length of stay. These two studies did not consider the presence of cirrhosis, and the other meta-analysis of non-cirrhotic cancer showed no difference in morbidity or the length of stay. A recent randomized controlled trial showed the effects of omega-3 fatty acids in reducing endotoxemia and sepsis in patients with chronic liver failure; however, it did not consider surgical patients [46]. This condition may generate hypotheses about the role of immunonutrients in patients with cirrhosis and inflammatory conditions.

Little research has been conducted regarding bladder surgery in recent years; however, immunonutrition has proven to be capable of inducing immunological changes and some reductions in postoperative complications. However, the Cochrane review showed that low-quality evidence was used due to the methodological limitations of these studies [22]. There is a lack of evidence regarding this type of surgery, and the study in question mainly included patients in a state of malnutrition before surgery, although there was no difference regarding the use of neoadjuvant chemotherapy or radiotherapy before surgery. Another limitation of these studies is the presence of an ERAS protocol, or lack thereof, in the study’s development [47].

Colorectal surgery is one of the main areas of immunonutrition research. In the last few years, the studies conducted regarding immunonutrition without an ERAS protocol have shown similar data to that collected in previous research, with a decrease in inflammatory markers and no effect on clinical features [23,24]. Recently, the potential use of immunonutrition in ERAS protocols has been studied, and it has been recommended by many societies as an intervention preceding this type of surgery to reduce the rate of patient complications and length of stay, especially in colorectal and duodenal-pancreatic surgery [47]. In many cases, it is used following a strong recommendation despite having a low level of evidence. To increase the amount of evidence, two multicenter studies provided immunonutrition to normally nourished patients in an ERAS protocol, and they observed a reduction in the rate of infectious complications [25,26]. These data are similar to those obtained in the meta-analysis performed by Xu et al. [27]. To achieve a better selection of patients, we need to determine the effects of these formulas on body composition and muscle function.

The use of immunonutrition has been most widely studied in the context of esophagogastric oncological surgery in the last few years, with many clinical trials and systematic reviews. Gastric and esophageal cancers cause a high rate of malnutrition, and the patients may need medical treatment for their nutrition before surgery to reduce the probability of complications [48]. Most studies showed a decrease in the infectious complications and inflammatory markers; however, the benefits regarding mortality and the length of stay remain unclear. These differences could be related to the localization of surgery (esophageal [35] or gastric [30]), the presence of neoadjuvant chemo- or radiotherapy, and the route of nutritional support (oral, gastrostomy, or jejunostomy) [36]. The most striking effects have been observed in patients who underwent immunonutrition treatment prior to surgery, and more studies are needed on immunonutrition via the jejunostomy route in order to make a conclusion on this topic. The increase in evidence regarding the use of these formulas for this type of patients has resulted in clinical guidelines recommending their use in upper digestive tract surgery [2]. Nevertheless, their use in an ERAS protocol is not yet fully recommended due to a lack of evidence in studies that included these protocols [49].

In pancreatic cancer, there are some deficiencies resulting from the type of surgery. There was a decrease in the rate infectious complications in all the studies [39,40,41]. However, there were no changes in the length of stay or morbidity. In all cases, the effect was better if immunonutrition was used before the surgery. In the case of a pathology with a high rate of malnutrition, it is necessary to differentiate the effect of increased intake versus that resulting from the use of immunonutrients added to oral nutritional supplements. There is a lack of evidence regarding the use of immunonutrition in ERAS protocols in pancreatic surgery [50].

The prognosis of tumor patients undergoing surgical treatment is closely related to the grade of tumor progression and neoadjuvant therapy (chemotherapy or radiotherapy) used to control the tumor before surgery. These conditions can influence nutritional status and the evolution of patient well-being in the perioperative period. The exclusion criteria of randomized clinical trials sometimes include the previous oncological treatment [14,16,24,31], type of histology [17,18,19,20,24,32], type of aggression, or timing from surgery [23,25,26,36]. However, systematic reviews and meta-analyses do not always take these factors into account, complicating the extrapolation of data and the adequate selection of patients to receive an immunonutrition treatment.

The immunonutrition treatment contents for surgical patients are variable, but there are some characteristics that are common to all these formulas, namely, an enrichment in amino acids such as arginine and/or glutamine, nucleotides, and different amounts of omega-3 fatty acids (enriched in EPA and DHA) (Supplementary Table S9). Nevertheless, it is difficult to identify the effect of every immunonutrient in formulas due to the high variability of these formulas, and no clear definition of the quantity of these nutrients has been created, although this is necessary to create adequate evidence regarding the effects of immunomodulatory nutrients.

The strengths of this study include the use of recent evidence to determine the potential uses of immunonutrition for surgical patients. Most of the studies are meta-analyses of previous high-quality studies, which allowed us to evaluate high-grade evidence. As we used a GRADE methodology to assess the studies, the information is of the best quality. This review contains a great body of evidence about patients who underwent oncologic surgery.

The main limitations of this review include the use of heterogeneous data from the studies, as they were selected to study a topic with broad contents. Oncologic surgical patients may have several different characteristics, and the effects of immunonutrition can change, making it difficult to determine the effects of these formulas. On the other hand, the term immunonutrition is very ambiguous and includes many different interventions and formulas, making it difficult to extrapolate the results. Some pathologies have little scientific evidence in general or from the period within the last seven years; therefore, we could not obtain valuable data.

Carrying out a wide-scope review of immunonutrition in surgical patients based on recent studies allowed us to determine the major deficits in the evidence for this topic. Future lines of investigation could include the relationship between the effects of immunonutrition on muscle mass and function after surgery in studies with morphofunctional assessments of disease-related malnutrition. Most existing studies are based on complications (overall and infectious); therefore, new studies should be designed to render the information about these formulas more complete, centering on nutritional status, hospital and community costs, or formula tolerance and adherence. More studies about the use of immunonutrition in ERAS protocols for different types of surgeries are also needed.

## 5. Conclusions

The use of immunonutrition, both pre- or postoperatively, in patients who underwent oncological surgery decreased the levels of inflammatory markers and infectious postoperative complications in almost all localizations. In the case of abdominal surgeries, immunonutrition can decrease the length of stay. The effect on mortality must be studied in more depth, controlling for confounding factors. Therefore, the proper selection of candidates for immunonutrition among patients undergoing oncological surgery is important and must be based on scientific evidence.

More studies are needed to assess the use of immunonutrition in ERAS protocols and in the evaluation of muscle mass and function from a morphofunctional point of view. In the same way, new studies should be designed to obtain more information about the effects of this type of formula on nutritional status, costs, and tolerance and adherence.

## Figures and Tables

**Table 1:** The search strategy design. Search strategy used to develop the systematic review.

Set	Items	Terms
S1	294	MJEMB(IMMUNONUTRITION)
S2	6759974	EMB(SURGERY) OR EMB(PREOPERATIVE PERIOD) OR EMB.EXPLODE(SURGERY) OR EMB(POSTOPERATIVE CARE) OR EMB(ENHANCED RECOVERY AFTER SURGERY) OR (“FAST TRACK SURGERY”) OR EMB.EXPLODE(POSTOPERATIVE COMPLICATION) OR (“POSTOPERATIVE MORBIDITY”) OR EMB(SURGICAL MORTALITY) OR EMB(HOSPITAL READMISSION) OR EMB.EXPLODE(MALIGNANT NEOPLASM) OR EMB(CANCER SURGERY)
S3	707888	EMB(TRAUMATIC BRAIN INJURY) OR EMB(SEPSIS) OR EMB(ACUTELY ILL PATIENT) OR EMB(BURN PATIENT) OR EMB(STEM CELL TRANSPLANTATION) OR EMB(BONE MARROW TRANSPLANTATION) OR EMB(INFLAMMATORY BOWEL DISEASE) OR EMB(CHRONIC INFLAMMATION)
S4	1557575	EMB(SARCOPENIA) OR (“SKELETAL MUSCLE MASS”) OR EMB(FUNCTIONAL STATUS) OR EMB(CACHEXIA) OR EMB(MALNUTRITION) OR EMB(BODY COMPOSITION) OR (“BIOELECTRICAL IMPEDANCE”) OR (“PHASE ANGLE”) OR EMB(ECHOGRAPHY) OR EMB(COMPUTER ASSISTED TOMOGRAPHY) OR (“L3 MUSCLE”) OR EMB(DUAL-ENERGY X-RAY ABSORPTIOMETRY)
S5	1361498	EMB(RANDOMIZED CONTROLLED TRIAL) OR EMB(SYSTEMATIC REVIEW) OR EMB(META ANALYSIS)
S6	7333881	EMB(HUMAN) AND (LA(ENGLISH) OR LA(SPANISH)) AND PY (≥2016)
S7	65	S1 AND (S2 OR S3 OR S4) AND S5 AND S6

**Table 2:** Summary of the evidence obtained based on the search strategy and selection of articles with evidence rating using GRADEpro for general oncological surgery. IN: immunonutrition; PO: postoperative; UTI: urinary tract infection.

Article	Study Type	Number of Patients	Type of Patients	Intervention	Results	GRADE
ONCOLOGIC SURGERY						
Yu K et al., 2020 [10]	Meta-analysis	5983 (61 studies)	Oncologic surgery	IN (oral, enteral, parenteral, at least one of the following: arginine, glutamine, w3, nucleotides) vs. SN, conventional therapy, or fluids in the pre-, peri-, and PO period.	Reduced total infectious complications (RR 0.71 (0.64, 0.79)), wound infection (*n* = 4788, RR 0.72 (0.60, 0.87)), respiratory infections (*n* = 4919, RR 0.70 (0.59, 0.84)), UTI (*n* = 3686, RR 0.69 (0.51, 0.94)), anastomotic dehiscence (*n* = 3329, RR 0.70 (0.53, 0.91)), and hospital LOS (–2.12 (–2.72, −1.52) days). No differences in sepsis (*n* = 2322) or overall mortality.	Low (++oo)
Buzquurz F et al., 2020 [11]	Meta-analysis	2159 (22 studies)	Oncologic surgery	IN from 30 days before surgery to at least 5 days before, which was allowed to continue after surgery, administered by oral or enteral route. The control group received standard care or placebo.	Reduced total infectious complications (*n* = 2068, RR 0.58 (0.48, 0.70)) and surgical site infection (*n* = 1958, RR 0.65 (0.50, 0.85)). No differences in mortality (*n* = 1641).	Moderate (+++o)

**Table 3:** Summary of the evidence obtained based on the search strategy and selection of articles with evidence rating using GRADEpro for head and neck surgery. IN: immunonutrition; SN: standard nutrition; ONS: oral nutrition supplement; PO: postoperative; LOS: length of stay.

Article	Study Type	Number of Patients	Type of Patients	Intervention	Results	GRADE
HEAD AND NECK CANCER						
Dechaphunkul T et al., 2022 [14]	RCT	110	Patients receiving chemoradiation	IN vs. isocaloric isonitrogenous formula 5 consecutive days before each chemotherapy session.	No difference in the proportion of patients with grade 3–4 oral mucositis between the two groups (62% vs. 67%, *p* = 0.690).	Low (++oo)
Howes N et al., 2018 [13]	Cochrane review	1099 (19 RCT)	Surgery for head and neck cancer	Pre-, peri- or PO IN vs. SN or no ONS.	No difference in hospital LOS, wound infection, or overall mortality. Reduced fistula: RR 0.48 (95% CI 0.27 to 0.85; 10 studies, 747 participants; low-quality evidence).	Moderate (+++o)

**Table 4:** Summary of the evidence obtained based on the search strategy and selection of articles with evidence rating using GRADEpro for hepatic surgery. IN: immunonutrition; SN: standard nutrition; ONS: oral nutrition supplement; PO: postoperative; LOS: length of stay.

Article	Study Type	Number of Patients	Type of Patients	Intervention	Results	GRADE
HEPATIC SURGERY						
Gao B et al., 2020 [15]	Meta-analysis	966 (9 studies)	Hepatectomy	Perioperative IN vs. SN, placebo or conventional diet (in 4 studies, ω-3 parenteral).	Reduced PO complications (RR 0.57 (0.34, 0.95)), total infections (RR 0.53 (0.37, 0.75)), wound infection (RR 0.50 (0.28, 0.89)), pneumonia (RR 0.60 (0.32, 1.12)), UTI (RR 1.30 (0.55, 3.08)), liver failure (RR 0.54 (0.23, 1.24)), mortality (RR 0.69 (0.26, 1.83)) and hospital LOS (–3.80 (–6.59, –1.02) days).	Low (++oo)
Zhang C et al., 2017 [16]	Meta-analysis	805 (8 studies)	Hepatectomy	Perioperative IN (in 6 studies, ω-3 enteral or parenteral) vs. isocaloric isonitrogenous nutrition	Reduced PO complications (RR 0.59 (0.46, 0.75)), total infections (RR 0.46 (0.32, 0.68)). No differences in mortality. Hospital stay was shorter in the ω-3 group (–0.49 (–0.81, –0.16) days).	Low (++oo)
Ciacio O et al., 2021 [17]	RCT	399	Liver resection for cancer (not cirrhosis)	Oral IN 7 days before vs. isocaloric isonitrogenous formula	No differences in 30-day morbidity rate (Clavien–Dindo >2), infectious and non-infectious complications, LOS, or duration of antibiotic treatment.	High (++++)
Uno H et al., 2016 [18]	RCT	40	Major hepatobiliary resection in cancer	Oral IN 5 days before (1000 kcal/day of IEN formula) vs. isocaloric diet	Reduced infections at 30 days (wound infection, abscesses, pneumonia, sepsis, 40 vs. 75% (*p* < 0.05)), shorter LOS (36.9 vs. 53.9 days, *p* < 0.01), lessened severity of complications (*p* < 0.05) (Clavien–Dindo scale), increased EPA/AA and resolvin E1, and decreased IL6 (same PCR results in both groups).	Moderate (+++o)

**Table 5:** Summary of the evidence obtained based on the search strategy and selection of articles with evidence rating using GRADEpro for bladder surgery. IN: immunonutrition; SN: standard nutrition; ONS: oral nutrition supplement; HC HP ONS: hypercaloric/hyperproteic oral nutritional supplement; PO: postoperative, UTI: urinary tract infection; LOS: length of stay.

Article	Study Type	Number of Patients	Type of Patients	Intervention	Results	GRADE
BLADDER SURGERY						
Hamilton-Reeves JM et al., 2018 [19]	RCT	29	Radical cystectomy (bladder cancer, not metastatic, not severe malnutrition)	Pre-IN (5 days) and post- IN (5 days) or HC HP ONS	Increased the T-helper lymphocytes (Th1–Th2) balance, decreased interleukin 6 (IL6), plasma arginine was maintained. No difference in appendicular muscle loss.	Low (++oo)
Hamilton-Reeves JM et al., 2016 [20]	RCT	29	Radical cystectomy (bladder cancer, not metastatic, no severe malnutrition)	Pre-IN (5 days) and post-IN (5 days) or HC HP ONS	33% reduction in PO complications at 90 days (RR 0.31, 95% CI 0.08 to 1.23, *p*:0.060), without differences in hospital LOS.	Low (++oo)
Alam SM et al., 2021 [21]	Systematic review	17 studies (6 with IN)	Radical cystectomy	2 studies compared IN with standard care and without ONS, 3 compared it with ONS, and in 1 the information was not available.	2 studies showed a reduction in PO complications, 1 study found immunological changes and a reduction in some inflammatory mediators, and 1 study found no differences in infectious complications. The other 2 studies are pending results.	Very Low (+ooo)
Burden S et al., 2019 [22]	Cochrane review	500 (8 RCT) IN 1 study with 29 patients	Radical cystectomy for bladder cancer	Perioperative nutrition	Limited evidence for a perioperative nutrition benefit from the interventions. IN reduced 90-day PO complications (RR 0.31, 95% CI 0.08 to 1.23; low-quality evidence). Similar hospital LOS.	Very low (+ooo)

**Table 6:** Summary of the evidence obtained based on the search strategy and selection of articles with evidence rating using GRADEpro for colorectal surgery. IN: immunonutrition; SN: standard nutrition; ONS: oral nutrition supplement; PO: postoperative; LOS: length of stay.

Article	Study Type	Number of Patients	Type of Patients	Intervention	Results	GRADE
COLORECTAL SURGERY						
Lee SY et al., 2021 [23]	RCT	176	Colon cancer	Preoperatory IN (400 mL/day) for 7 days vs. standard diet.	No differences in PO infectious and noninfectious complications, overall complications, 30-day readmission, hospital LOS, or weight.	Moderate (+++o)
Wierdak M et al., 2021 [24]	RCT	26	Colorectal cancer	14 days before surgery IN (400 mL/day) vs. ONS.	Changed inflammatory response ((tumoral necrosis factor-α) TNF-α, interleukin 8 (IL-8), C-X-C Motif Chemokine Ligand 1 (CXCL1)), superficial neutrophil infiltration. No changes in morbidity, LOS, or readmissions.	Moderate (+++o)
Moya P et al., 2016 [25]	RCT	122	Laparoscopic colorectal resection (not malnourish)	IN (400 mL/day) for 7 days before and 5 days after surgery vs. dietetic recommendations.	Reduced cases of wound infection (11.50 vs. 0.00%, *p* = 0.006). No differences in hospital LOS.	Moderate (+++o)
Moya P et al., 2016 [26]	RCT	244	Colorectal resection (not malnourished)	IN (400 mL/day) for 7 days before and 5 days after surgery vs. isocaloric isonitrogenous formula.	Reduced overall complications, mainly due to a decrease in the number of infectious complications (23.8% vs. 0.7%, *p* = 0.0007). Surgical site infection number was lower (16.4% vs. 5.7%, *p* = 0.0008). No differences in hospital LOS.	Moderate (+++o)
Xu J et al., 2018 [27]	Systematic Review	6 Studies with enteral immunonutrition	Colorectal surgery	RCT Enteral Immunonutrition vs Standard formula	Immunonutrition can reduce the length of hospital stay (OR 2.53 (1.29–2.41)) and number of infectious complications (OR 0.33; (0.21–0.53))	Low (++oo)

**Table 7:** Summary of the evidence obtained based on the search strategy and selection of articles with evidence rating using GRADEpro for esophagogastric surgery. IN: immunonutrition; SN: standard nutrition; ONS: oral nutrition supplement; PO: postoperative, UTI: urinary tract infection; LOS: length of stay.

Article	Study Type	Number of Patients	Type of Patients	Intervention	Results	GRADE
ESOPHAGOGASTRIC SURGERY						
Cao Y et al., 2022 [29]	Meta-analysis	1864 (15 studies, RCT and observational)	Esophageal cancer	Preoperatory IN vs. isocaloric isonitrogenous formula	Reduced infectious complications (OR = 0.51, 95% CI (0.26, 0.98) and length of hospital stay (MD = −2.10 days), 95% CI (−3.72, −0.47)). No differences in overall complications, in-hospital mortality, or anastomotic leaks.	Very Low (+ooo)
Cheng Y et al., 2018 [30]	Meta-analysis	583 (7 studies)	Gastric cancer	PO IN vs. EN	Increased levels of CD4^+^, CD4^+^/ CD8^+^, IgM, IgG, lymphocytes, and prealbumin (when administered for >7 days), reduced systemic inflammatory response syndrome (MD, –0.89 days; 95% CI, –1.40 to–0.39; *p* = 0.005) and postoperative complications (RR, 0.29; 95% CI, 0.14–0.60; *p* = 0.001). Pulmonary infection, length of hospitalization, CD8^+^, and other serum proteins were not improved.	Low (++oo)
Niu JW et al., 2021 [31]	Meta-analysis	16 studies	Gastrointestinal cancer	Perioperative IN vs. SN	Decreased the risk of surgical site infection, hospital stay (in addition to early enteral nutrition after surgical resection of gastric cancer), WBC, and CRP. No changes in CD4^+^ or inflammatory cytokines.	Low (++oo)
Adiamah et al., 2021 [32]	RCT	108	Esophagogastric or pancreaticobiliary cancer (not metastatic nor chemotherapy)	PO (10–15 days), jejunostomy, IN vs. isocaloric isonitrogenous formula	No differences in long-term survival	High (++++)
Li XK et al., 2020 [33]	Meta-analysis	320 (6 studies)	Esophageal cancer	Perioperative IN vs. SN	No improved clinical outcomes or immune indices.	High (++++)
Zhuo ZG et al., 2021 [34]	Meta-analysis	638 (6 studies)	Esophageal cancer	Perioperative IN vs. SN.	No significant differences in PO complications.	High (++++)
Li XK et al., 2021 [35]	RCT	112	Esophageal cancer	Preoperatory (7 days) IN vs. oral nutrition and PO jejunostomy EIN vs. EN.	Reduced the rate of CD8/CD3 at POD 3, increased the rate of CD4/CD8 at POD 3, IgM at POD 3 and 7, and the rates of NK and IgA at PDD 30. No significant differences in 2-year progression-free survival or overall survival.	Moderate (+++o)
Kanekiyo S et al., 2019 [36]	RCT	40	Esophageal cancer	Preoperatory (7 days) IN vs. oral nutrition and PO (7 days) jejunostomy EIN vs. EN.	Increased RBP, decreased infectious complications and changes to therapeutic antibiotics. No differences in ICU or hospital LOS, 5-year progression-free survival, or overall survival.	High (++++)
Song GM et al., 2017 [37]	Meta-analysis	840 (11 RCT)	Gastric cancer	Perioperative IN vs. SN. Comparison of different IN formulas.	Decreased IC and LOS. Arg+RNA+ω-3-FAs was superior to Arg+RNA and Arg+Gln for IC.	Moderate (+++o)
Wong CS et al., 2016 [38]	Meta-analysis	2016 (19 RCT)	Upper gastrointestinal surgery	Perioperative IN vs. SN.	Reduced wound infections (RR 0.59, 95% CI 0.40–0.88; *p* = 0.009) and hospital LOS (MD −2.92 days, 95% CI −3.89 to −1.95; *p* < 0.00001).	Moderate (+++o)

**Table 8:** Summary of the evidence obtained based on the search strategy and selection of articles with evidence rating using GRADEpro for pancreatic surgery. IN: immunonutrition; SN: standard nutrition; ONS: oral nutrition supplement; PO: postoperative; LOS: length of stay.

Article	Study Type	Number of Patients	Type of Patients	Intervention	Results	GRADE
PANCREATIC SURGERY						
Takagi K et al., 2020 [39]	Meta-analysis	349 (5 studies)	Partial pancreatoduodenectomy	IN vs. SN, PN, or no intervention	Reduced overall PO complications and infections. No effects on major complications, mortality, fistula, or delayed gastric emptying.	Moderate (+++o)
Guan H et al., 2019 [40]	Meta-analysis	299 (4 RCT)	Pancreatoduodenectomy	Perioperative IN vs. SN.	Reduced PO infectious complications (RR 0.58, 95% CI 0.37–0.92; *p* = 0.02) and hospital LOS (MD −1.79, 95% CI −3.40 to 0.18; *p* = 0.03). No differences in overall PO complications, non-infectious complications, or PO mortality.	Moderate (+++o)
Yang F et al., 2020 [41]	Meta-analysis	368 (6 RCT)	Pancreatic cancer	Perioperative IN vs. SN.	Decreased the rate of infectious complications (RR = 0.47, 95% CI (0.23, 0.94), *p* = 0.03) and the hospital LOS (MD = −1.90, 95% CI (−3.78, −0.02), *p* = 0.05), especially in the preoperative group.	High (++++)

**Table 9:** Main results of the effects of immunonutrition in review studies based on the function and localization of surgery.

Localization of Surgery	Effect of Immunonutrition	Level of Evidence
General oncologic	Decreased infectious complications and length of stay	MODERATE
Head and neck	Fistula formation	MODERATE
Esophagogastric surgery	Decreased infectious complications and length of stay	MODERATE
Colorectal surgery	Immunological changes, decreased wound infections and length of stay	MODERATE
Hepatic surgery	Decreased infectious complications, liver failure, mortality, and length of stay	MODERATE
Pancreatic surgery	Decreased infectious complications and length of stay	MODERATE
Bladder surgery	Immunological changes	LOW

## Data Availability

Not applicable.

## Supplementary Materials

The following supporting information can be downloaded at: https://www.mdpi.com/article/10.3390/nu15071776/s1, Table S1: PRISMA checklist for systematic review; Table S2: GradePro table for oncologic surgery; Table S3: GradePro table for head and neck cancer surgery; Table S4: GradePro table for hepatectomy; Table S5: GradePro table for bladder cancer; Table S6: GradePro table for colorectal cancer surgery; Table S7: GradePro table for esophagogastric cancer surgery; Table S8: GradePro table for pancreas cancer surgery; Table S9: Details from Randomized Clinical Trials included in the review.
