# Supplementary material for: Effects of Immunonutrition on Cancer Patients Undergoing Surgery: A Scoping Review
Source: Nutrients. 2023 Apr 5;15(7):1776. doi: 10.3390/nu15071776 (PMC10096769; doi:10.3390/nu15071776)
Supplement: Supplementary file 1 [file nutrients-15-01776-s001.zip › S2 oncologic surgery.pdf]

Author(s):
Question: Immunonutrition compared to standard nutrition in surgery of oncologic patients
Setting:
Bibliography:

Certainty assessment							№ of patients		Effect		Certainty	Importance
№ of studies	Study design	Risk of bias	Inconsistency	Indirectness	Imprecision	Other considerations	immunonutrition	standard nutrition	Relative (95% CI)	Absolute (95% CI)		
**Complications, mortality and length of hospital stay (Yu K, et al. 2020).**												
61	randomised trials	serious[1,a]	not serious	not serious	serious[a]	none	-/3186	-/2797	not estimable		⨁⨁◯◯ Low	
**Infectious complications and mortality (Buzquurz F, et al. 2020).**												
22	randomised trials	serious[2,b]	not serious	not serious	not serious	none			not estimable		⨁⨁⨁◯ Moderate	

CI: confidence interval

Explanations

a. Many studies have unclear risk of bias. There is 1 study with a high risk of bias in randomization. There are 4 studies with error in achieving blinding and blinding cannot be ensured in 4 studies when filling in the clinical results. In 3 studies, a high risk of error was detected when reporting outcomes due to the fact that there are many missing data. Outcomes were not well defined in 1 study. In 8 studies there are other types of bias because they were financed by laboratories. Data for the primary endpoint were reported in 48 studies, so the number of patients studied for each outcome varies. GRADE between low-moderate. Heterogeneity of the studies in terms of type and stages of cancer, long period of time analyzed, heterogeneity in terms of average stay (not other items). Inclusion of different nutrition schemes in terms of route, moment, duration, type of immunonutrients.
b. Patients with stage IV cancer were excluded. Immunonutrition is defined as that which has 2 or more of the following components: arginine, glutamine, w3, RNA or nucleotides. 12 studies administer perioperative nutrition (10 post nutrition by tube and 2 orally), 9 pre and 3 peri and pre. The duration of follow-up was variable between studies. In 6 studies, nutritional assessment was not carried out and it was heterogeneous according to the studies. Compliance with immunonutrition is described in 12 studies. In all studies except one, immunonutrition was tolerated the same as the control group. GRADE scale: it has been necessary to lower a level.

References

1.Yu K, Zheng X,Wang G,Liu M,Li Y,Yu P,et al. Immunonutrition vs Standard Nutrition for Cancer Patients: A Systematic Review and Meta-Analysis (Part 1). JPEN J Parenter Enteral Nutr 2020, https://doi.org/10.1002/jpen.1736., 44:742–67.. .
2.Buzquurz F, Bojesen RD,Grube C,Madsen MT,Gögenur I. Impact of oral preoperative and perioperative immunonutrition on postoperative infection and mortality in patients undergoing cancer surgery: systematic review and meta-analysis with trial sequential analysis. BJS Open 2020, https://doi.org/10.1002/bjs5.50314., 4:764–75.. .
