# Supplementary material for: Effects of Immunonutrition on Cancer Patients Undergoing Surgery: A Scoping Review
Source: Nutrients. 2023 Apr 5;15(7):1776. doi: 10.3390/nu15071776 (PMC10096769; doi:10.3390/nu15071776)
Supplement: Supplementary file 1 [file nutrients-15-01776-s001.zip › S3 Head and Neck Cancer.pdf]

Author(s):
Question: Inmunonutrition compared to standard formula for head and neck cancer
Setting:
Bibliography:

Certainty assessment							Impact	Certainty	Importance
№ of studies	Study design	Risk of bias	Inconsistency	Indirectness	Imprecision	Other considerations			
**Dechaphunkul, 2022 mucositis prevention[1]**									
1	randomised trials	serious	not serious	serious	not serious	none		⨁⨁◯◯ Low	
**Howes, 2018[2]**									
16	randomised trials	serious	not serious	not serious	not serious	none		⨁⨁⨁◯ Moderate	

CI: confidence interval

References

1.Dechaphunkul T, et al.. Benefits of immunonutrition in patients with head and neck cancer receiving chemoradiation: A phase II randomized, double-blind study. Clinical Nutrition 41 (2022) 433-440; 2022.
2.Howes, . Immunonutrition for patients undergoing surgery for head and neck cancer. Cochrane Database Syst Rev. 2018 Aug 30;8(8):CD010954;
