# Supplementary material for: Effects of Immunonutrition on Cancer Patients Undergoing Surgery: A Scoping Review
Source: Nutrients. 2023 Apr 5;15(7):1776. doi: 10.3390/nu15071776 (PMC10096769; doi:10.3390/nu15071776)
Supplement: Supplementary file 1 [file nutrients-15-01776-s001.zip › S4 Hepatectomy.pdf]

Author(s):
Question: Immunonutrition compared to conventional nutrition for hepatectomy because of cancer
Setting:
Bibliography:

Certainty assessment							№ of patients		Effect		Certainty	Importance
№ of studies	Study design	Risk of bias	Inconsistency	Indirectness	Imprecision	Other considerations	immunonutrition	conventional nutrition	Relative (95% CI)	Absolute (95% CI)		
**Postoperative complications, mortality and length of stay (Gao B, et al. 2020).**												
9	randomised trials	serious[1,a]	not serious	serious[a]	not serious	none	-/485	-/481	not estimable		⊕⊕◯◯ Low	
**30 day morbidity, complications, length of hospital stay, antibiotic treatment (Ciacio O, et al. 2021).**												
1	randomised trials	not serious[2]	not serious	not serious	not serious	none	-/199	-/200	not estimable		⊕⊕⊕⊕ High	
**Resolvin E1 (Uno H, et al. 2016).**												
1	randomised trials	not serious[3]	not serious	not serious	serious[b]	none	-/20	-/20	not estimable		⊕⊕⊕◯ Moderate	
**Postoperative complications, mortality, length of hospital stay (Zhang C, et al. 2017).**												
8	randomised trials	not serious[4]	serious[c]	serious[d]	not serious	none	-/402	-/403	not estimable		⊕⊕◯◯ Low	

CI: confidence interval

Explanations

a. The benefits observed in the intervention group may be due to the effect of macronutrient and vitamin and mineral supplementation rather than immunonutrients.
b. 2 studies with pre-surgery nutrition (minimum 5 days), 5 post-surgery (5-7 days) and 1 perioperative (7 days pre and 3 days post). Immunonutrition was in 6 studies w3 parenteral or enteral and in 2 studies with Impact. The control was isocaloric diet in all studies and protein control was similar in 3 studies.
c. The sample size of several studies is small. There are heterogeneity in diagnoses and nutrition administration routes. Nutritional assessment was not performed. There are heterogeneous definitions of clinical outcomes.
d. Only 9 studies, only in English, heterogeneity in the introduced population and in the immunonutrition schemes and there are only clinical data, not laboratory data. 4 studies compare immunonutrition with standard nutrition, 3 with placebo and 2 with conventional diet. 4 with Impact pre-surgery, 1 with enteral w3 pre-surgery and 4 with parenteral w3 (3 post-surgery), most of the studies during 5 days.

References

1.Gao B, Luo J,Liu Y,Zhong F,Yang X,Gan Y,et al. Clinical Efficacy of Perioperative Immunonutrition Containing Ome-ga-3-Fatty Acids in Patients Undergoing Hepatectomy: A Systematic Review and Meta-Analysis of Randomized Controlled Trials. Ann Nutr Metab 2020, https://doi.org/10.1159/000509979., 76:375–86.. .
2.Ciacio O, Voron T,Pittau G,Lewin M,Vibert E,Adam R,et al. Interest of preoperative immunonutrition in liver resection for cancer: study protocol of the PROPILS trial,a multicenter randomized controlled phase IV trial. BMC Cancer 2014, https://doi.org/10.1186/1471-2407-14-980., 14:980.. .
3.Uno H, Furukawa K,Suzuki D,Shimizu H,Ohtsuka M,Kato A,et al. Immunonutrition suppresses acute inflammatory re-sponses through modulation of resolvin E1 in patients undergoing major hepatobiliary resection. Surgery 2016, https://doi.org/10.1016/j.surg.2016.01.019., 160:228–36.. .
4.Zhang C, Chen B,Jiao A,Li F,Wang B,Sun N,et al. The benefit of immunonutrition in patients undergoing hepatectomy: a systematic review and meta-analysis. Oncotarget 2017, https://doi.org/10.18632/oncotarget.20045., 8:86843–52.. .
