# Supplementary material for: Effects of Immunonutrition on Cancer Patients Undergoing Surgery: A Scoping Review
Source: Nutrients. 2023 Apr 5;15(7):1776. doi: 10.3390/nu15071776 (PMC10096769; doi:10.3390/nu15071776)
Supplement: Supplementary file 1 [file nutrients-15-01776-s001.zip › S5 Bladder.pdf]

Author(s):
Question: Immunonutrition compared to standard nutrition for cistectomy for bladder cancer
Setting:
Bibliography:

Certainty assessment							№ of patients		Effect		Certainty	Importance
№ of studies	Study design	Risk of bias	Inconsistency	Indirectness	Imprecision	Other considerations	immunonutrition	standard nutrition	Relative (95% CI)	Absolute (95% CI)		
**Hamilton-Reeves JM et al. 2018. Th1-Th2 balance, IL6, plasma arginine, appendicular muscle loss.**												
1	randomised trials	serious[1,a]	not serious	not serious	serious[a]	none	-/14	-/15	not estimable		⊕⊕◯◯ Low	
**Hamilton-Reeves JM et al. 2016. Postoperative complications at 90 days and hospital length of stay.**												
1	randomised trials	serious[2,b]	not serious	not serious	serious[b]	none	-/14	-/15	not estimable		⊕⊕◯◯ Low	
**Alam SM, et al. 2021. Postoperative complications, immunological changes and inflammatory mediators.**												
6	observational studies	serious[3,c]	not serious	serious[d]	serious[e]	none			not estimable		⊕◯◯◯ Very low	
**Burden S et al. 2019. Postoperative complications and hospital length of stay.**												
1	randomised trials	serious[4,f]	not serious	serious[g]	serious[g]	none			not estimable		⊕◯◯◯ Very low	

CI: confidence interval

Explanations

a. Small sample size and low participation on days 14 and 30 of the postoperative period.
b. Small sample size. Imprecision of design and results (according to Cochrane).
c. 17 articles (only 6 related to immunonutrition, 2 of them abstracts that are in the recruitment period or in progress. 2 studies compare immunonutrition with standard care without nutritional oral supplement (NOS), 3 with NOS and in 1 the information is not available. In 2 studies Immunonutrition (N;14) has been randomized with standard NOS (N;15), in another 2 studies immunonutrition has been compared with historical controls and another 2 studies are in the recruitment period or pending results.
d. 2 studies compare immunonutrition with standard care without NOS, 3 with NOS and in 1 the information is not available.
e. 2 studies show a reduction in postoperative complications, another study found immunological changes and a reduction in some inflammatory mediators, and 1 study found no differences in infectious complications. The other 2 studies are pending results.
f. 29 patients.
g. Limited evidence for a benefit of perioperative nutrition interventions. Immunonutrition reduced 90-day postoperative complications (RR 0.31, 95% CI 0.08 to 1.23; low-quality evidence). Similar hospital length of stay.

References

1.Hamilton-Reeves JM, Stanley A,Bechtel MD,Yankee TM,Chalise P,Hand LK,et al. Perioperative Immunonutrition Modu-lates Inflammatory Response after Radical Cystectomy: Results of a Pilot Randomized Controlled Clinical Trial. J Urol 2018, https://doi.org/10.1016/j.juro.2018.03.001., 200:292–301.. .
2.Hamilton-Reeves JM, Bechtel MD,Hand LK,Schleper A,Yankee TM,Chalise P,et al. Effects of Immunonutrition for Cystec-tomy on Immune Response and Infection Rates: A Pilot Randomized Controlled Clinical Trial. Eur Urol 2016, https://doi.org/10.1016/j.eururo.2015.11.019., 69:389–92.. .
3.Alam SM, Michel C,Robertson H,Camargo JT,Linares B,Holzbeierlein J,et al. Optimizing Nutritional Status in Patients Undergoing Radical Cystectomy: A Systematic Scoping Review. Bladder Cancer Amst Neth 2021, https://doi.org/10.3233/blc-200428., 7:449–61.. .
4.Burden S, Billson HA,Lal S,Owen KA,Muneer A. Perioperative nutrition for the treatment of bladder cancer by radical cystectomy. Cochrane Database Syst Rev 2019, https://doi.org/10.1002/14651858.CD010127.pub2., 5:CD010127.. .
