# Supplementary material for: Effects of Immunonutrition on Cancer Patients Undergoing Surgery: A Scoping Review
Source: Nutrients. 2023 Apr 5;15(7):1776. doi: 10.3390/nu15071776 (PMC10096769; doi:10.3390/nu15071776)
Supplement: Supplementary file 1 [file nutrients-15-01776-s001.zip › S6 Colorectal.pdf]

Author(s):
Question: Immunonutrition compared to standard nutrition for colorectal cancer surgery
Setting:
Bibliography:

Certainty assessment							№ of patients		Effect		Certainty	Importance
№ of studies	Study design	Risk of bias	Inconsistency	Indirectness	Imprecision	Other considerations	immunonutrition	standard nutrition	Relative (95% CI)	Absolute (95% CI)		
**Lee SY et al. 2021. Complications, 30-day readmission, hospital length of stay and weight.**												
1	randomised trials	serious[1,a]	not serious	not serious	not serious	none	-/88	-/88	not estimable		⨁⨁⨁◯ Moderate	
**Moya P et al. 2016. Wound infection and length of stay.**												
1	randomised trials	serious[2,b]	not serious	not serious	not serious	none	-/61	-/61	not estimable		⨁⨁⨁◯ Moderate	
**Moya P et al. 2016. Complications and length of stay.**												
1	randomised trials	serious[3,b]	not serious	not serious	not serious	none	-/122	-/122	not estimable		⨁⨁⨁◯ Moderate	
**Wierdak M et al. 2021. Inflammatory response (TNF-α, CXCL8, CXCL1, superficial neutrophil infiltration), morbidity, length of stay or readmissions.**												
1	randomised trials	serious[4,c]	not serious	not serious	not serious	none	-/14	-/12	not estimable		⨁⨁⨁◯ Moderate	
**Xu J et al. 2017. Length of hospital stay, infections and inflammatory molecules.**												
9	randomised trials	serious[d]	not serious	serious[e]	not serious	none	-/502	-/502	not estimable		⨁⨁◯◯ Low	

CI: confidence interval

Explanations

a. Most of the included patients were non-obese, well-nourished patients. Small number of malnourished patients (8.5%). Adherence to nutritional supplements was not evaluated.
b. Malnourished patients were excluded.
c. Small number of patients included.
d. Three studies did not give the sufficient information about randomization. Blinding of participants and personnel was conducted in four studies.
e. More studies with specific timings (preoperative, perioperative, and postperative) are needed for better understanding of immunonutrition in clinical practice.

References

1.Lee SY, Lee J,Park H-M,Kim CH,Kim HR. Impact of Preoperative Immunonutrition on the Outcomes of Colon Cancer Sur-gery: Results from a Randomized Controlled Trial. Ann Surg 2021. https://doi.org/10.1097/SLA.0000000000005140.. .
2.Moya P, Soriano-Irigaray L,Ramirez JM,Garcea A,Blasco O,Blanco FJ,et al. Perioperative Standard Oral Nutrition Sup-plements Versus Immunonutrition in Patients Undergoing Colorectal Resection in an Enhanced Recovery (ERAS) Protocol: A Multicenter Randomized Clinical Trial (SONVI Study). Medicine (Baltimore) 2016, https://doi.org/10.1097/MD.0000000000003704., 95:e3704.. .
3.Moya P, Miranda E,Soriano-Irigaray L,Arroyo A,Aguilar M-D-M,Bellón M,et al. Perioperative immunonutrition in nor-mo-nourished patients undergoing laparoscopic colorectal resection. Surg Endosc 2016, https://doi.org/10.1007/s00464-016-4836-7., 30:4946–53.. .
4.Wierdak M, Surmiak M,Milian-Ciesielska K,Rubinkiewicz M,Rzepa A,Wysocki M,et al. Immunonutrition Changes In-flammatory Response in Colorectal Cancer: Results from a Pilot Randomized Clinical Trial. Cancers 2021, https://doi.org/10.3390/cancers13061444., 13:1444.. .
