# Supplementary material for: Effects of Immunonutrition on Cancer Patients Undergoing Surgery: A Scoping Review
Source: Nutrients. 2023 Apr 5;15(7):1776. doi: 10.3390/nu15071776 (PMC10096769; doi:10.3390/nu15071776)
Supplement: Supplementary file 1 [file nutrients-15-01776-s001.zip › S7 Esophagogastric.pdf]

Author(s):
Question: Inmunonutrition compared to standard formula in upper gastrointestinal surgery
Setting:
Bibliography: [1]

Certainty assessment							Impact	Certainty	Importance
**№ of studies**	**Study design**	**Risk of bias**	**Inconsistency**	**Indirectness**	**Imprecision**	**Other considerations**			
**Song, 2017[2]**									
11	randomised trials	not serious	not serious	not serious	serious[a]	none		⨁⨁⨁◯ Moderate	
**Zuho 2021[3]**									
6	randomised trials	not serious	not serious	not serious	not serious	none		⨁⨁⨁⨁ High	
**Wong CS [4]**									
19	randomised trials	serious	not serious	not serious	not serious	none		⨁⨁⨁◯ Moderate	
**Adiamah 2021[5]**									
1	randomised trials	not serious	not serious	not serious	not serious	none		⨁⨁⨁⨁ High	
**Cao 2022 [6]**									
15	randomised trials	serious	serious[b]	not serious	serious[c]	none		⨁◯◯◯ Very low	
**Cheng 2018[7]**									
7	randomised trials	serious	not serious	not serious	serious[d]	none		⨁⨁◯◯ Low	
**Li 2020[8]**									
1	randomised trials	not serious	not serious	not serious	not serious	none		⨁⨁⨁⨁ High	
**Niu JW 2021[9]**									
6	randomised trials	serious[e]	serious[f]	not serious	not serious	none		⨁⨁◯◯ Low	
**Li XK 2021[10]**									
1	randomised trials	not serious	serious[g]	not serious	not serious	none		⨁⨁⨁◯ Moderate	
**Kanekiyo, 2019[11]**									
1	randomised trials	not serious	not serious	not serious	not serious	none		⨁⨁⨁⨁ High	

CI: confidence interval

Explanations

a. Regional data bases
b. Observational studies
c. lack time with IN
d.
e. Half of studies from same country
f. no exlusion criteria
g. laboratory values without clinical relevance

References

1.Yu J, et al.. Impact of perioperative enteral immunonutrition in patients with gastrointestinal cancer undergoing elective surgery: a randomized controlled trial.. Clinical Nutrition ESPEN; 2021.
2.Song, . Systematic review with network meta-analysis: comparative efficacy of different enteral immunonutrition formulas in patients underwent gastrectomy. Oncotarget. ; 2017.
3.Zhuo, G. Z. et al. Is immunonutrition superior to standard enteral nutrition in reducing postoperative complications in patients undergoing esophagectomy? A meta-analysis of randomized controlled trials. BUON 2021; 26(1): 204-210; 2021.

4.Wong CS, Aly EH. The effects of enteral immunonutrition in upper gastrointestinal surgery: A systematic review and meta-analysis. International Journal of Surgery 29 (2016) 137e150; 2016.
5.A, Adiamah. Postoperative arginine-enriched immune modulating nutrition:Long-term survival results from a randomised clinical trial in patients with oesophagogastric and pancreaticobiliary cancer. Clinical Nutrition 40 (2021) 5482e5485; 2021.
6.Cao, . Effects of preoperative nutrition on postoperative outcomes in esophageal cancer:a systematic review and meta-analysis. Diseases of the Esophagus (2022)35,1–13; 2022.
7.Chen, . Enteral immunonutrition versus enteral nutrition for gastric cancer patients undergoing a total gastrectomy: a systematic review and meta-analysis. BMC Gastroenterology (2018) 18:11; 2018.
8.Ka Li, Yujie Xu,Yanjie Hu,Yuwei Liu,Xinrong Chen & Yong Zhou. Effect of Enteral Immunonutrition on Immune, Inflammatory Markers and Nutritional Status in Gastric Cancer Patients Undergoing Gastrectomy: A Randomized Double-Blinded Controlled Trial. Journal of Investigative Surgery, DOI: 10.1080/08941939.2019.1569736; 2019.
9.. . Niu JW, Zhou L, Liu ZZ, Pei DP, Fan WQ, Ning W. A Systematic Review and Meta-Analysis of the Effects of Perioperative Immunonutrition in Gastrointestinal Cancer Patients. Nutr Cancer. 2021;73(2):252-261;
10.Li XK, Cong ZZ,Wu WJ,Xu Y,Zhou H,Wang GM,Qiang Y,Luo LG,Shen Y. Enteral immunonutrition versus enteral nutrition for patients undergoing esophagectomy: a randomized controlled trial. Ann Palliat Med. 2021 Feb, 10(2):1351-1361, . .
11.Kanekiyo S, Takeda S,Iida M,Nishiyama M,Kitahara M,Shindo Y,Tokumitsu Y,Tomochika S,Tsunedomi R,Suzuki N,Abe T,Yoshino S,Hazama S,Ueno T,Nagano H. Efficacy of perioperative immunonutrition in esophageal cancer patients undergoing esophagectomy. Nutrition. 2019 Mar, 59:96-102., . .
