# Supplementary material for: Effects of Immunonutrition on Cancer Patients Undergoing Surgery: A Scoping Review
Source: Nutrients. 2023 Apr 5;15(7):1776. doi: 10.3390/nu15071776 (PMC10096769; doi:10.3390/nu15071776)
Supplement: Supplementary file 1 [file nutrients-15-01776-s001.zip › S8 Pancreas.pdf]

Author(s):
Question: Inmunonutrition compared to standard formula in pancreatic surgery
Setting:
Bibliography: [1,2]

Certainty assessment							№ of patients		Effect		Certainty	Importance
№ of studies	Study design	Risk of bias	Inconsistency	Indirectness	Imprecision	Other considerations	inmunonutrition	standard formula	Relative (95% CI)	Absolute (95% CI)		
**Tagaki 2020, postsurgical complications (assessed with: riesgo relativo)**												
5	randomised trials	serious	not serious	not serious	not serious	none	46/107 (43.0%)	98/175 (56.0%)	**RR 0.74** (0.58 to 0.94)	**146 fewer per 1000** (from 235 fewer to 34 fewer)	⊕⊕⊕◯ Moderate	IMPORTANT
**Tagaki 2020, major complications (assessed with: RR)**												
5	randomised trials	serious[a]	not serious	not serious	not serious	none	24/107 (22.4%)	49/175 (28.0%)	**RR 0.68** (0.41 to 1.12)	**90 fewer per 1000** (from 165 fewer to 34 more)	⊕⊕⊕◯ Moderate	IMPORTANT
**Tagaki 2020, mortality (assessed with: RR)**												
5	randomised trials	serious[a]	not serious	not serious	not serious	none	2/127 (1.6%)	5/185 (2.7%)	**RR 0.79** (0.16 to 3.99)	**6 fewer per 1000** (from 23 fewer to 81 more)	⊕⊕⊕◯ Moderate	IMPORTANT
**Tagaki 2020, infectious complications (assessed with: RR)**												
5	randomised trials	serious	not serious	not serious	not serious	none	31/144 (21.5%)	60/205 (29.3%)	**RR 0.60** (0.42 to 0.84)	**117 fewer per 1000** (from 170 fewer to 47 fewer)	⊕⊕⊕◯ Moderate	IMPORTANT
**Yang 2020, general complications (assessed with: RR)**												
6	randomised trials	not serious	not serious	not serious	not serious	none	35/104 (33.7%)	46/108 (42.6%)	**RR 0.79** (0.50 to 0.12)	**89 fewer per 1000** (from 375 fewer to 213 fewer)	⊕⊕⊕⊕ High	IMPORTANT
**Yang 2020, infectious complications**												
6	randomised trials	not serious	not serious	not serious	not serious	none	16/129 (12.4%)	32/133 (24.1%)	**RR 0.50** (0.30 to 0.84)	**120 fewer per 1000** (from 168 fewer to 38 fewer)	⊕⊕⊕⊕ High	IMPORTANT
**Yang 2020 mortality (assessed with: RR)**												
6	randomised trials	not serious	not serious	not serious	not serious	none	3/148 (2.0%)	2/149 (1.3%)	**RR 1.35** (0.27 to 6.88)	**5 more per 1000** (from 10 fewer to 79 more)	⊕⊕⊕⊕ High	IMPORTANT
**Yang 2020 long of stay**												
6	randomised trials	not serious	not serious	not serious	not serious	none	LOS MD = −2.03; 95% CI = −3.78, −0.45; p = 0.01)				⊕⊕⊕⊕ High	IMPORTANT
**Guan 2019 general complications (assessed with: RR)[3]**												
4	randomised trials	serious	not serious	not serious	not serious	none	51/129 (39.5%)	65/133 (48.9%)	**RR 0.81** (0.62 to 1.05)	**93 fewer per 1000** (from 186 fewer to 24 more)	⊕⊕⊕◯ Moderate	IMPORTANT
**Guan 2019, infectious complications**												

4	randomised trials	serious	not serious	not serious	not serious	none	21/146 (14.4%)	52/153 (34.0%)	**RR 0.58** (0.37 to 0.92)	**143 fewer per 1000** (from 214 fewer to 27 fewer)	⨁⨁⨁◯ Moderate	IMPORTANT
**Guan 2019, mortality**												
4	randomised trials	serious	not serious	not serious	not serious	none	3/129 (2.3%)	1/133 (0.8%)	**RR 2.43** (0.37 to 16.10)	**11 more per 1000** (from 5 fewer to 114 more)	⨁⨁⨁◯ Moderate	IMPORTANT

CI: confidence interval; **RR:** risk ratio

Explanations

a. Risk of vias

References

1.Takagi, . . Langenbecks Arch Surg (2020) 405:585–593;
2.Yang F, et al.. Immunonutrition in Patients with Pancreatic Cancer Undergoing Surgical Intervention: A Systematic Review and Meta-Analysis of Randomized Controlled Trials. Nutrients ; 2020, .
3.Guan H, et al. Effects of Enteral Immunonutrition in Patients Undergoing Pancreaticoduodenectomy: A Meta-Analysis of Randomized Controlled Trials. Ann Nutr Metab 2019;74:53–61;
