# Supplementary material for: Effects of Immunonutrition on Cancer Patients Undergoing Surgery: A Scoping Review
Source: Nutrients. 2023 Apr 5;15(7):1776. doi: 10.3390/nu15071776 (PMC10096769; doi:10.3390/nu15071776)
Supplement: Supplementary file 1 [file nutrients-15-01776-s001.zip › Supplementary Data S9.pdf]

Supplementary Materials. S9: Details of RCTs

Study	Study design	Number of patients	Main objective	Secondary objectives	Exclusion criteria	Type of intervention	Results	Limitations
**HEAD AND NECK CANCER**								
Dechaphunkul T et al, 2022 [14]	RCT	110 (55 in intervention group, 55 in control group)	To compare the effect of immunonutrition with placebo in the prevention of mucositis.	Effects of immunonutrition on other complications of chemoradiotherapy, nutritional status, tolerance to treatment and survival	Curative surgery for the treatment of head and neck cancer or allergy to any of the components of immunonutrition	Administration of 303 kcal of immunomodulatory formula (w3 fatty acids, nucleotides and arginine) or standard formula, 3 times a day during the 5 days prior to the treatment session	There were no differences in grade II and IV mucositis between groups (62% in the experimental group vs 66% in the control group p=0.690). There were also no differences in terms of nutritional parameters. There were no differences in overall survival at 3 years (69 vs 50% HR 0.60). If there were differences in disease-free survival at 3 years 69% vs 44% (p=0.056)	Survival analysis was not the main objective and the record of compliance was that reported by the patient without objective verification methods. In addition, in the experimental group there were more patients who received intensity-modulated radiotherapy.
**HEPATIC SURGERY**								
Ciacio O et al, 2021 [17]	RCT<br>Multicentric<br>6 centers in France	399 (199 intervention group, 200 control group)	Impact of preoperative immunonutrition in patients undergoing liver resection for cancer (non-cirrhotic) on 30-day morbidity.	Infectious and non-infectious morbidity at 30 days, time of antibiotic treatment, average hospital length of stay, treatment compliance and adverse effects.	Cirrhosis of the liver, biliary or gastrointestinal surgery accompanying hepatectomy, hemodialysis, pregnancy, a history of hypersensitivity to the components of immunonutrition,	7 days before surgery Impact® or Impact control® (isocaloric formula with the same protein intake without immunonutrients) 909 kcal and 50.4 g of protein/day.	The 30-day morbidity rate (Clavien-Dindo >2) was similar in both groups (44.7 vs 44.9%, p=0.954). Also, infectious (21 vs 18%, p=0.498) and non-infectious (38 vs 39%, p=0.7) complications, mean stay (12 vs 11 days,	In the publication on the study methodology, there are more secondary objectives: stay in the ICU, changes in intake, liver regeneration (by estimating liver volume with CT), sarcopenia (by

					inability to take nutrition, and mental illness that makes it impossible to understand the objectives of the study.		p=0.803) and antibiotic treatment time (2.7 vs 2.5 days, p=0.598).	measuring the psoas at L3-L4) and economic analysis.
Uno H et al, 2016 [49]	RCT Unicentric Japan	40 (20 intervention group, 20 control group)	Effect of immunonutrition on resolvin E1 levels in patients after major hepatobiliary resection for cancer	Effect of immunonutrition on postoperative results in patients after major hepatobiliary resection for cancer	<18 years, >80 years, active infection, GI obstruction, respiratory, cardiac, hepatic, renal dysfunction, immunocompromised disease including QT, RT, metastatic disease	5 days before the surgery 1000 kcal/day of oral Impact® (NOS enriched with EPA, arginine and nucleotides). To do this, the intake of conventional foods was reduced by 50%. The control group had a 2000 kcal diet without NOS. The patient was hospitalized to monitor intake. Food intake was allowed on 3 postoperative day	Less infections at 30 days (wound infection, abscesses, pneumonia, sepsis) 40 vs 75% (p<0.05), shorter mean stay (36.9 vs 53.9 days, p<0.01), less severity of complications (p<0.05) (Clavien-Dindo scale), increased EPA/AA, resolvin E1, and decreased IL6 (same PCR in both groups)	Small number of patients, the benefits observed in the intervention group may be due to the effect of macronutrient and vitamin and mineral supplementation rather than immunonutrients. Nutritional assessment is not performed.
**BLADDER SURGERY**								
Hamilton-Reeves JM et al. 2018 [18]	RCT (unicentric, USA)	29 (14 in intervention group, 15 in control group)	To evaluate the impact of the use of perioperative immunonutrition on IL-6 levels and the Thelper1-Thelper2 balance in patients	Effect of immunonutrition on nutritional status, physical activity and quality of life in patients after radical cystectomy	Patients with swallowing disorder, metastatic disease, food allergies, active viral infection, history of uric lithiasis or gouty arthritis.	The intervention group took 3 bricks/day of Impact® 5 days before the surgery and 5 days after the radical cystectomy between meals. The control group took 3 bricks/day of an SNO (Boost plus®) 5	An increase in the Th1-Th2 balance (TNF-alpha:IL-13 ratio) on day 2 postoperative in the intervention group versus a decrease in the control group (p < 0.027). IL-6 levels significantly lower in the	Small sample size and low participation on days 14 and 30 of the postoperative period. Patients with severe malnutrition, BMI < 18.5 kg/m² and those who have lost > 10% of

			undergoing radical cystectomy			days before the surgery and 5 days after the radical cystectomy between meals.	immunonutrition group (p 0.020) than in the control group. No differences in body weight. Less loss of muscle mass in the intervention group but without statistical significance. No changes in quality of life or functional	weight in the last 6 months are excluded.
Hamilton-Reeves JM et al, 2018 [19].	RCT (unicentric, USA)	29 (14 in intervention group, 15 in control group)	To evaluate the impact of the use of perioperative immunonutritio n on postoperative complications in patients undergoing radical cystectomy	No other secondary objectives are described	Patients with swallowing disorder, metastatic disease, food allergies, active viral infection, history of uric lithiasis or gouty arthritis.	The intervention group took 3 bricks/day of Impact® 5 days before the surgery and 5 days after the radical cystectomy between meals. The control group took 3 bricks/day of an SNO (Boost plus®) 5 days before the surgery and 5 days after the radical cystectomy between meals.	In the intervention group, a 33% reduction in postoperative complications was achieved at 90 days (RR 0.31, 95% CI 0.08 to 1.23, p:0.060), without differences in hospital stay	Imprecision of design and results (according to Cochrane). Small sample size and low participation on days 14 and 30 of the postoperative period. Patients with severe malnutrition, BMI < 18.5 kg/m^2 and those who have lost > 10% of weight in the last 6 months are excluded.
**COLORECTAL SURGERY**								
Lee SY et al, 2021 [21]	RCT, unicentric, South Korea	176	To evaluate the impact of the use of preoperative immunonutritio n on the rate of postoperative infectious	To assess the impact of preoperative immunonutrition on the rate of postoperative complications, change in body weight, and length of hospital stay	Emergency surgery, difficulty in oral intake, pregnancy and planned ostomy	The intervention group was taking 400ml/day of a protein, arginine and w-3 rich NOS, in addition to the normal diet for 7 consecutive days	There were no differences in relation to infectious complications between the immunonutrition group (17.7%) and control group	Most of the included patients were non-obese, normally nourished patients (8.5% malnutrition); adherence to the

			complications in patients with colon neoplasia			before surgery. The patients in the group controls ate a normal diet without administering a placebo.	(15.9%) (P = 0.751), nor in total postoperative complications (31.6% vs 29.3%, P = 0.743) nor in non-infectious complications (15.2% vs. 18.3%, P = 0.598). The readmission rate at 30 days did not show significant differences between the two groups (2.5% vs. 6.1%, p = 0.443) nor the prolongation of hospital stay (7.6 ± 2.5 vs. 7.4 ± 2.3 days, P = 0.635). the change in body weight was similar in both groups.	use of nutritional supplements was not evaluated.
Moya P et al, 2016 [23]	RCT, unicentric, Spain	122 (61 in intervention group, 61 in control group)	To determine the effect exerted by the simultaneous implementation of immunonutritio n and the laparoscopic approach in morbidity, mortality and length of hospital stay compared with	Secondary objectives are not described	Patients <18 years old, malnourished, laparotomy, urgent surgery, ASA IV, hemodialysis, taking NOS, impossibility for oral intake, pregnancy, psychiatric illnesses, HIV, sepsis, intestinal obstruction.	The intervention group took 400ml/day of an NOS enriched in arginine, nucleotides and w-3, in addition to the normal diet for 7 days before surgery and 5 days after surgery. Control group patients only received dietary advice	Immunonutrition significantly reduced wound infection (11.50 vs. 0.00%, p = 0.006). There were no significant differences in relation to hospital stay between the 2 groups.	Only normally-nourished patients, immunonutrition is not compared with other NOS, possible confounding factors are not considered.

			no nutritional supplementation in patients with colon cancer					
Moya P et al, 2016. [24]	RCT, unicentric, Spain	244 (122 in intervention group, 122 in control group)	To examine whether the co-implementation of immunonutrition together with an enhanced recovery after surgery (ERAS) program improves morbidity, mortality and length of stay compared to using a standard NOS.	Secondary objectives are not described	Patients <18 years old, malnourished, laparotomy, urgent surgery, ASA IV, hemodialysis, taking NOS, impossibility for oral intake, pregnancy, psychiatric illnesses, HIV, sepsis, intestinal obstruction.	The immunonutrition group took 400ml/day of an NOS enriched in arginine, nucleotides and w-3, in addition to the normal diet for 7 days before and 5 days after surgery. The control group received 400ml/day of a hypercaloric and hyperproteic NOS along with diet for 7 days before and 5 days after surgery.	Reduction in total complications in the immunonutrition group compared to the control group, mainly due to a decrease of infectious complications (23.8% vs 0.7%, P = 0.0007). Among the infectious complications, surgical site infection was significantly lower in the intervention group (16.4% vs. 5.7%, P = 0.0008). There were no significant differences in relation to hospital stay between the 2 groups.	Baseline infection rates in the 2 groups higher than those of other hospitals/authors. Normally nourished patients, possible confounding factors are not considered.
Wierdak M et al. [22]	RCT, unicentric, Poland	26 (14 in intervention group, 12 in control group)	To evaluate the impact of the use of immunonutrition in the preoperative period on the inflammatory	Secondary objectives are not described	Histopathologic diagnosis other than adenocarcinoma, emergency surgery, active intestinal infection, other	The immunonutrition group was administered 400ml/day of an NOS enriched in arginine, nucleotides and w-3, for 14 days	TNF-alpha expression was different after surgery between the two groups (immunonutrition group	Low sample size. The authors comment that these results are demonstrated at the molecular and microscopic level that they do not

			response, the expression of cytokines (TNF, IL-8, chemokines) and the infiltration of leukocytes in the tumor tissue in patients undergoing surgery for colorectal cancer.		systemic immunological disorder, need for preoperative neoadjuvant therapy (radiotherapy or chemotherapy), metastatic disease, <85% NOS intake.	before surgery. The control group received 600ml/day of a standard NOS for the same time.	31.63 ± 13.28; control group 21.54 ± 6.84; p = 0.049). There were changes in CXCL8 expression in the control group before and after surgery (before: 2975.93 ± 1484.04; after: 1584.85 ± 1659.84; p = 0.041). The expression of CXCL1 was increased in the immunonutrition group and decreased in the control (immunonutrition 2698.27 (1538.14–5124.70); control 953.75 (457.85–1534.60); p = 0.032). In both groups there was a decrease in the superficial infiltration of neutrophils (being significant in the immunonutrition group). There were no significant differences between the 2 groups in relation to mortality, morbidity, hospital stay or readmissions.	have evidence that the observed changes had a clinically significant impact on tumor biology and the course of neoplastic disease.
**ESOFAGOGASTRIC SURGERY**								

Adiamah A et al, 2021 [30].	RCT, 3 hospitals in United Kingdom	108 (54 in intervention group and 54 in control group)	Analyzes the long-term survival (20 years) of patients included in a previous RCT to determine if arginine supplementation, as part of jejunostomy immunonutrition, in the postoperative period for esophagogastric or pancreatic cancer improves survival. compared to isocaloric and isoproteic nutrition in the control group	Secondary objectives are not described	Metastatic or unresectable disease, pregnancy, prior chemotherapy	Jejunostomy nutrition started 4 hours after surgery at 25 ml/h, 50 ml/h on day 1 postoperative and 75 ml/h the following days, for 20 hours a day, for a period of 10-15 days after surgery. Group A receives the experimental formula (Stresson® Nutricia) and Group B an isonitrogen and isocaloric formula (Nutrison High protein®).	They find no significant differences in long-term survival	The experimental formula contains, in addition to triple arginine, more glutamine, cysteine and w-3, slightly less total fat and more Na and K than the control group.
Li X-K et al, 2021 [33].	RCT	112	To investigate the efficacy of preoperative and postoperative enteral immunonutrition versus enteral nutrition on clinical and immunological outcomes of	2-year progression free survival and overall survival.	Inability to swallow; a history of allergy to milk or soy; acute or unstable cardiac conditions (e.g., unstable angina or symptomatic severe aortic stenosis); cardiac failure (New York Heart Association functional	The immunonutrition and enteral nutrition were commenced on pre-operative day 7 by 500 mL/day via oral intake with energy of 750 kcal/day in addition to diet before surgery. Jejunal enteral feeding was commenced on	Enteral immunonutrition yielded a significantly lower rate of CD8/CD3 (%) at postoperative day 3 compared with enteral nutrition group (P=0.005). The rate of CD4/CD8 (%) in enteral immunonutrition	It changes inflammation parameters without effect on clinical variables.

			patients undergoing esophagectomy. Immune indicator was the primary outcome in this study		classes III and IV); severe renal dysfunction, chronic obstructive pulmonary disease (forced expiratory volume in the first second of expiration <60% predicted); liver dysfunction, or acute pancreatitis.	postoperative day 1 at 20–25 mL/hour. Oral feeding resumed following bedside assessment of swallow for patients who underwent esophagectomy with cervical anastomosis at postoperative day 5, meanwhile liquid diet was implemented by oral intake with 100–200 g. From that time, patients recommenced a graded introduction to diet from sips to free fluids, light diet (soup/jelly/ice-cream) by day 6. Patients of home enteral nutrition were instructed to independently administer jejunostomy feeding at home if discharge and jejunostomy tubes were removed at post-discharge day (PDD) 30 uniformly. The patients were regarded as lost for follow-up, if their jejunostomy tubes	group was higher than that in enteral nutrition group at postoperative day 3 (P=0.004). The serum levels of IgM at postoperative day 3 and 7 were significantly higher in enteral nutrition group compared with immunonutrition enteral group (P=0.025 and P=0.009, respectively). The rate of NK (%) and the serum level of IgA were significantly higher in immunonutrition enteral group compared with enteral nutrition group at PDD 30 (P=0.022 and P=0.041, respectively). No significant differences were found in 2-year progression free survival and overall survival.	

						were removed after surgery and before PDD 30 due to complications or other reasons.		
Kanekiyo S et al [34].	RCT, single center	40	Number of infective complications (including changes to therapeutic antibiotics)	Nutritional status, postoperative intensive care unit (ICU) stay, postoperative hospital length of stay, another clinical outcome, and long-term outcome.	Patients who underwent thoracoscopic and/or laparoscopic procedures	The patients were assigned randomly to receive immunomodulating enteral nutrition with either Impact® or Ensure®. The patients received enteral nutrition associated with their regular meals for 7 consecutive days before surgery, and postoperative enteral nutrition through a jejunostomy tube placed during surgery for 7 days after surgery.	Levels of retinol-binding protein were significantly higher on postoperative day (POD) −1, POD 7, and POD 14 in the intervention compared with the standard group ($p=0.009$, $p=0.004$, and $p=0.024$, respectively). The incidence of postoperative infectious complications and changes to therapeutic antibiotics were significantly lower in the intervention compared with the standard group ($p=0.048$ and $p=0.012$, respectively). There was no significant difference in postoperative ICU or postoperative hospital length of stay between the two groups. The 5-	Not found.

							year progression-free survival rates in the intervention and standard groups were 75% and 64%, respectively (p=0.188), and the overall survival rates were 68% and 55%, respectively (p=0.187).	
